# Gene expression of hepatic gluconeogenic and fatty acid metabolism in early-lactation dairy cows as affected by dietary starch and monensin supplementation

**DOI:** 10.3168/jdsc.2023-0430

**Published:** 2023-11-04

**Authors:** M.M. McCarthy, G.D. Mechor, A.W. Holloway, T.R. Overton, E.A. Horst

**Affiliations:** ^1^ Department of Animal Science, Cornell University, Ithaca, NY 14853; ^2^ Elanco Animal Health, Greenfield, IN 46140

## Abstract

Our previously published companion papers demonstrated improved production performance and energetic metabolism in cows fed diets with increased propiogenic potential in early lactation. Study objectives were to further explore effects of dietary starch content and monensin on hepatic gene expression of key enzymes related to gluconeogenesis and fatty acid metabolism in early lactation. From d 1 to 21 postpartum, primiparous (n = 16) and multiparous (n = 33) Holstein cows were fed a high (HS; 26.2% starch, 34.3% neutral detergent fiber, 22.7% acid detergent fiber, 15.5% crude protein) or low (LS; 21.5% starch, 36.9% neutral detergent fiber, 25.2% acid detergent fiber, 15.4% crude protein) starch diet with a daily topdress containing either 0 (Con) or 450 mg/d monensin (Mon). Cows were randomly assigned to treatment. Liver biopsies were obtained from cows on d 7 postpartum for DNA and RNA quantification and mRNA expression analysis. In primiparous cows, Mon supplementation decreased *CPT1A* expression relative to controls, whereas in multiparous cows Mon increased its expression. Cows fed HS and Mon tended to have decreased *HMGCS2* expression relative to cows fed HS and Con. In multiparous cows, Mon supplementation tended to increase *PC* and *PCK1* expression relative to controls. Correlation analysis was performed for all gene expression variables. Overall, relationships were similar in directionality and magnitude between cows fed HS and LS and Con and Mon. However, for cows fed Con there was a positive relationship between *HMGCS2* and *PC* and *HMGCS2* and *PCK1*, whereas for cows fed Mon there was no relationship. There was a similar lack of relationship between *HMGCS2* and *PC* for cows fed HS. Overall, results support changes in performance and energetic metabolism reported in our companion papers, indicating that cows fed diets of different starch content in early lactation with Mon supplementation throughout the transition period had alterations in hepatic gene expression consistent with increased hepatic propionate supply.

## Highlights

• Monensin increased hepatic gluconeogenic capacity in multiparous cows, as shown by increased pyruvate carboxylase and cytosolic phosphoenolpyruvate gene expression, independently of dietary starch level.
• Monensin increased TCA cycle flux, as shown by increased carnitine palmitoyl-transferase 1A expression in multiparous cows.
• Monensin decreased ketogenesis, as shown by decreased 3-hydroxy-3-methyl-glutaryl-CoA synthase gene expression in cows fed a high-starch diet postpartum.
• Changes in hepatic gene expression are consistent with previously reported changes in circulating metabolites.

## Graphical Abstract

**Figure:**
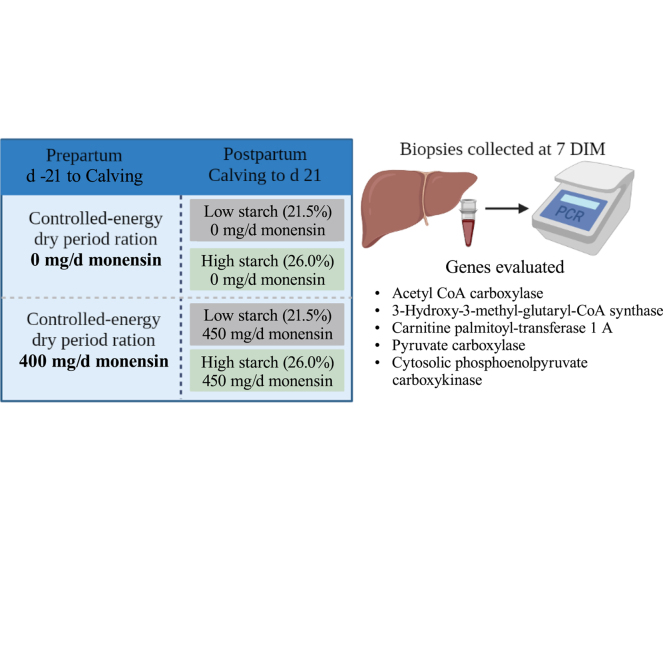
**Summary** This study assessed the effects of dietary starch level and monensin supplementation on hepatic expression of genes related to gluconeogenesis and fatty acid metabolism in postpartum dairy cows. From day 1 to 21 postpartum, primiparous (n = 16) and multiparous (n = 33) Holstein cows were fed a high- or low-starch diet with a daily topdress containing either 0 mg/day (control) or 450 mg/day monensin. Liver biopsies were obtained from cows on day 7 postpartum for DNA and RNA quantification and mRNA expression analysis. Overall, results indicated that monensin supplementation had minor effects on genes related to liver gluconeogenic capacity, tricarboxylic acid (TCA) cycle flux, and ketogenesis, whereas starch level had little to no effect.

Glucose supply in early lactation is crucial to supporting milk production (Neville, 1990). Propionate, derived from ruminal starch degradation, is the primary substrate for hepatic gluconeogenesis in a ruminant and its supply can be independently increased by increasing dietary starch level or feeding monensin (Drackley et al., 2001; McCarthy et al., 2015a,b). Hepatic conversion of propionate to glucose is responsive to propionate supply (Zhang et al., 2016) and there is a positive linear relationship between propionate and liver glucose output (Dijkstra et al., 2005). In our companion publications (McCarthy et al., 2015a,b), we demonstrated improved postpartum performance and energetic metabolism with increased dietary starch content postpartum and monensin supplementation throughout the periparturient period, although the effects of starch level and monensin were independent of each other (McCarthy et al., 2015a,b). Study objectives herein were to determine whether changes in energetic metabolism were reflected by altered expression of genes involved in hepatic gluconeogenesis and fatty acid oxidation at d 7 postpartum. We hypothesized that increasing dietary starch content postpartum and feeding monensin throughout the periparturient period would increase expression of genes related to gluconeogenesis and fatty acid oxidation.

All animal procedures were approved by the Cornell University Institutional Animal Care and Use Committee (#2012–0035), and the experiment was conducted from March to October 2012. The experimental design and treatments were described completely in previous publications (McCarthy et al., 2015a,b). Briefly, a 2 × 2 factorial arrangement of postpartum treatments was used with early-lactation period feeding strategy (high-starch [**HS**] vs. low-starch [**LS**] diet during the first 21 d postpartum) and monensin supplementation (0 mg of monensin/d [**Con**] or 450 mg of monensin/d [**Mon**]; monensin; Elanco Animal Health, Greenfield, IN) as the variables of interest. Cows were randomly assigned to treatment. Liver tissue was sampled via percutaneous trocar biopsy (Veenhuizen et al., 1991) from a subset of cows (primiparous, n = 16; multiparous, n = 33) under local anesthesia on d 7 (±4 d range; ±1.6 SD) postpartum. After blotting the liver tissue to remove excess blood and connective tissue, the sample was snap-frozen in liquid nitrogen and stored at −80°C until analysis for DNA and RNA quantification.

To quantify DNA and RNA, 150 μg of liver tissue was homogenized in 5 mL of DNA assay buffer (50 m*M* Na_2_PO_4_, 2 *M* NaCl, 2 m*M* Na_2_EDTA). Hoechst 33258 dye binding was used for DNA quantification (Labarca and Paigen, 1980). A standard curve was prepared containing 0 to 2 μg of calf thymus DNA (Sigma Aldrich, St. Louis, MO) per well. Triplicate 5-µL aliquots of sample homogenates were transferred to a 96-well microplate. Then 195 μL of DNA assay buffer containing Hoechst dye (99.8 mL of DNA assay buffer + 200 μL of dye solution [1 μg of Hoechst 33258 dye/μL of distilled water]) was added to each sample. Fluorescence was read with a SpectraMax M2 plate reader with 360/460 nm filter set (Molecular Devices, Sunnyvale, CA). A second 0.5-mL aliquot of the above liver tissue homogenate was placed in a 2-mL microcentrifuge tube and RNA was determined by UV absorbance using the method described by Schmidt and Thannhauser (1945). Absorbance was measured at 260 and 232 nm with a SpectraMax 190 plate reader (Molecular Devices).

Total RNA was isolated and purified using miRNeasy minicolumns and on-column ribonuclease-free deoxyribonuclease treatment (Qiagen Inc., Valencia, CA). Quantity and integrity of RNA was determined using the RNA Nano Lab chip kit and Bioanalyzer (Agilent, Palo Alto, CA). Reverse transcriptase reactions were performed with 2 µg of RNA in a 20-µL volume using the high-capacity cDNA reverse transcription kit (Applied Biosystems, Foster City, CA) according to the manufacturer's recommendation. Real-time PCR assays were performed in duplicate in a 20-µL volume using predesigned TaqMan primer probes for acetyl-CoA carboxylase α (*ACACA*), 3-hydroxy-3-methyl-glutaryl-CoA synthase (*HMGCS2*), pyruvate carboxylase (*PC*), and cytosolic phosphoenolpyruvate carboxykinase (*PCK1*), with the exception of carnitine palmitoyl-transferase 1A (*CPT1A*), which was a custom-designed primer probe (Applied Biosystems). All primer probes spanned exons. Reactions contained 500 n*M* of each primer (TaqMan probes) and diluted cDNA (20 ng). An arbitrary expression level was obtained by using the ΔΔC_t_ method. A calibrator sample was run for all genes and expression of TATA-box binding protein (*TBP*) was used as the invariant control. The C_t_ for the gene of interest in the test samples was adjusted in relation to expression of *TBP* (ΔC_t sample_), and the gene of interest C_t_ for calibrator samples was also adjusted in relation to expression of *TBP* (ΔC_t calibrator_). Any mRNA with a cycle number greater than 36 was declared undetectable. All gene expression results are expressed as relative abundance.

Statistical computations were performed using SAS software (version 9.3, SAS Institute Inc., Cary, NC). Data for DNA quantification, RNA quantification, and gene expression were subjected to ANOVA using the MIXED procedure of SAS. Fixed effects included starch level, monensin treatment, parity, and all 2-way interactions. The random effect was cow nested within starch and monensin treatment. Least squares means and standard error of the mean are reported. Statistical significance was declared at *P* ≤ 0.05. Because a subset of cows was used for all liver analyses, trends were declared at 0.05 < *P* < 0.15. This tendency threshold was also used in our companion paper for in vitro liver metabolism results (McCarthy et al., 2015b). Relationships between data for gene expression were analyzed using the PROC CORR statement and Pearson option in SAS.

Results from hepatic DNA and RNA quantification are presented in Table 1. In agreement with our previously reported production and metabolism results (McCarthy et al., 2015a,b), few starch by Mon interactions were observed on hepatic gene expression. The DNA content tended to be increased in primiparous cows fed LS relative to HS diets (8.77 vs. 7.34 mg DNA/g tissue; *P* = 0.11; Table 1), which may reflect increased cell number and liver mass (Baldwin et al., 2004). However, no effect of starch level on DNA content was observed in multiparous cows (7.67 vs. 7.79 mg of DNA/g of tissue). Relative to LS, HS cows tended to have increased RNA content (*P* = 0.13), which may indicate an increase in protein synthesis capacity (Baldwin et al., 2004). No treatment differences were detected in the RNA:DNA, which provides an index of protein synthesis capacity per cell (Wagner et al., 1998).

**Table 1:** Postpartum hepatic DNA, RNA content, and mRNA expression on d 7 (±1.6 SD) postpartum for cows fed either high- or low-starch diets and control or monensin treatments in early lactation

Item	Diet^1^		SEM	Topdress^2^		SEM	*P*-value^3^					
	HS	LS		Con	Mon		S	M	P	S × M	S × P	M × P
DNA, mg/g tissue	7.57	8.22	0.35	8.07	7.72	0.35	0.17	0.46	0.50	0.38	0.11	0.96
RNA, mg/g tissue	9.64	9.14	0.24	9.54	9.24	0.24	0.13	0.38	0.72	0.72	0.50	0.79
RNA:DNA ratio	1.32	1.16	0.08	1.25	1.23	0.08	0.17	0.86	0.51	0.76	0.22	0.94
FA synthesis-related gene expression^4^												
*ACACA*	1.20	1.18	0.11	1.15	1.23	0.12	0.93	0.63	0.04	0.14	0.82	0.61
FA oxidation-related gene expression^5^												
*CPT1A*	0.83	0.75	0.04	0.80	0.78	0.04	0.23	0.84	0.83	0.87	0.78	0.13
Ketogenesis-related gene expression^6^												
*HMGCS2*	0.71	0.77	0.06	0.80	0.69	0.06	0.44	0.22	0.64	0.12	0.39	0.61
Gluconeogenesis-related gene expression^7^												
*PC*	0.52	0.45	0.04	0.46	0.51	0.05	0.32	0.36	0.52	0.39	0.66	0.13
*PCK1*	0.98	0.84	0.08	0.85	0.97	0.08	0.24	0.32	0.97	0.69	0.84	0.09

1 HS = high-starch diet (26.2% starch); LS = low-starch diet (21.5% starch).

2 Con = control topdress, formulated to supplement 0 mg/d monensin; Mon = monensin topdress, formulated to supplement 450 mg/d postpartum.

3 S = starch; M = monensin; P = parity.

4 *ACACA* = acetyl-CoA carboxylase α. FA = fatty acid.

5 *CPT1A* = carnitine palmitoyl-transferase 1A.

6 *HMGCS2* = 3-hydroxy-3-methyl-glutaryl-CoA synthase.

7 *PC* = pyruvate carboxylase; *PCK1* = cytosolic phosphoenolpyruvate carboxykinase.

Relative to Con, Mon supplementation increased *CPT1A* expression in multiparous cows (0.84 vs. 0.76; *P* = 0.13), which agrees with others (Mullins et al., 2012) and presumably reflects improved fatty acid oxidation capacity. However, the opposite response was detected in primiparous cows (0.73 vs. 0.84, in Mon and Con, respectively). Andersen et al. (2002) observed that cows fed more propiogenic diets had increased fatty acid oxidation and less liver triglyceride infiltration. Propionate carbon enters the tricarboxylic acid (**TCA**) cycle at succinyl-CoA and is eventually converted to oxaloacetate (**OAA**; Figure 1). During periods of increased glucose demand, OAA removal from the TCA cycle is increased to support gluconeogenesis (Aschenbach et al., 2010). This cataplerotic reaction results in decreased availability of OAA to condense with acetyl-CoA culminating in reduced TCA cycle flux and diversion of acetyl-CoA toward ketogenesis and liver triglyceride synthesis (Krebs, 1966). Increasing propionate supply likely alleviates depletion of the OAA pool, which may allow for enhanced oxidation of acetyl-CoA during periods of increased gluconeogenesis. While ATP demands for gluconeogenesis may be sufficiently met via increased β-oxidation, enhanced TCA flux likely still occurs with increased propionate supply and is supported in the current study by changes in *HMGCS2* and circulating BHB concentrations as described below.

**Figure 1:**
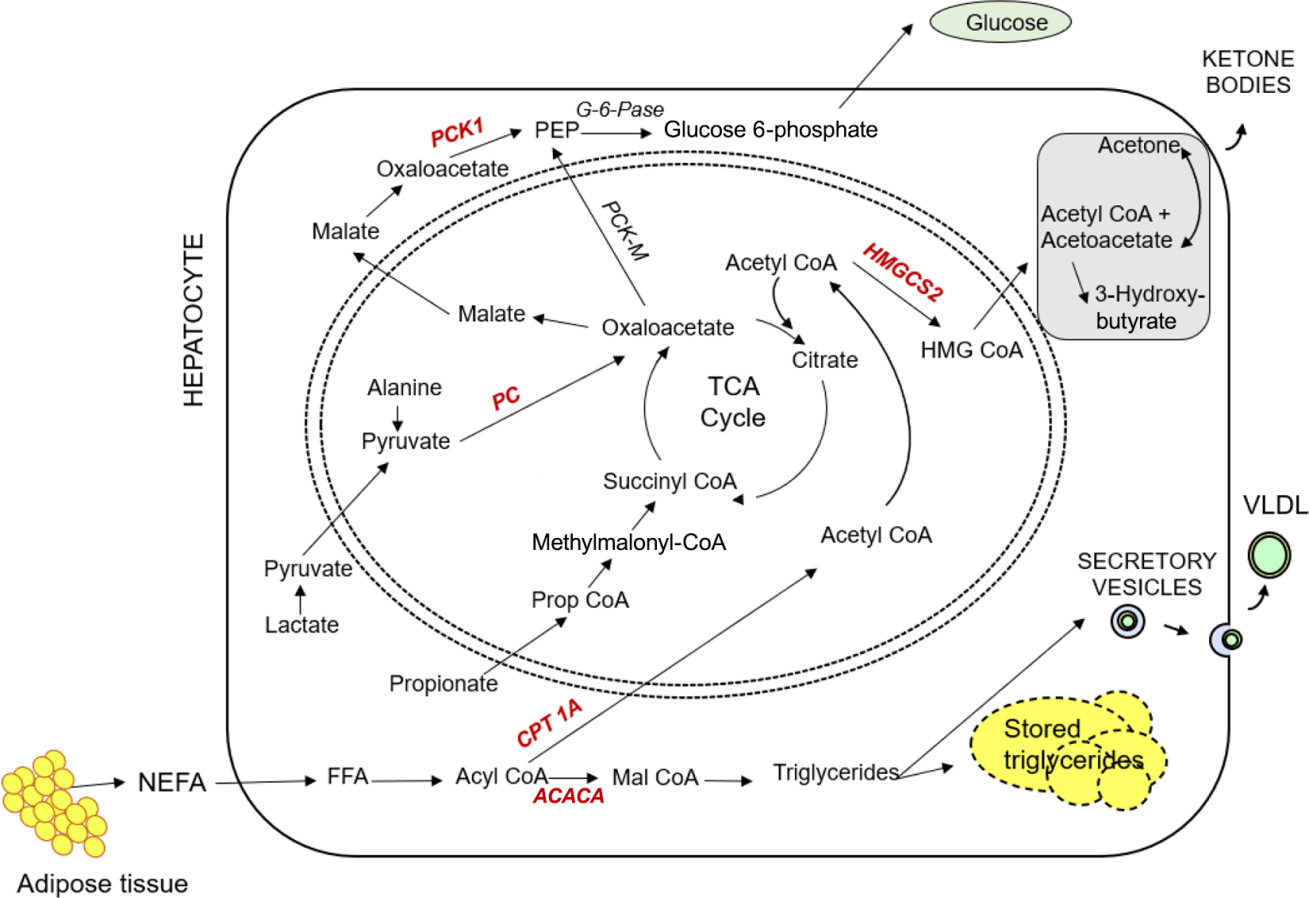
Key gene-encoding enzymes of the gluconeogenesis, ketogenesis, and fatty acid metabolism pathways: acetyl-CoA carboxylase α (*ACACA*), carnitine palmitoyl-transferase 1A (*CPT1A*), 3-hydroxy-3-methyl-glutaryl-CoA synthase (*HMGCS2*), pyruvate carboxylase (*PC*), cytosolic phosphoenolpyruvate carboxykinase (*PCK1*). G-6-Pase = glucose 6-phosphatase; PEP = phosphoenolpyruvate; HMG CoA = 3-hydroxy-3-methylglutaryl coenzyme; PCK-M = mitochondrial phosphoenolpyruvate carboxykinase; Prop CoA = propionyl coenzyme; mal CoA = malonyl coenzyme; FFA = free fatty acid; NEFA = nonesterified fatty acid; TCA = tricarboxylic acid; VLDL = very-low-density lipoprotein.

In agreement with increased *CPT1A* expression, Mon tended to decrease *HMGCS2* abundance relative to Con in cows fed HS diets (*P* = 0.12; 0.59 vs. 0.83). The intramitochondrial activity of *HMGCS2* is the rate-limiting step in converting acetyl-CoA to ketone bodies (Drackley et al., 2001) and conversion of succinyl-CoA to succinate leads to inhibition of its expression (Zammit, 1990). Combined effects of Mon on *CPT1A* and *HMGCS2* expression are consistent with propionate serving as both a cataplerotic and anaplerotic metabolite. In addition, these results support findings from our companion publication, which demonstrated decreased circulating BHB concentrations with Mon supplementation (McCarthy et al., 2015b).

One of the key enzymes in hepatic de novo fatty acid synthesis is *ACACA* (Vernon, 2005; Figure 1). Although the absolute activity of hepatic *ACACA* in ruminants is substantially lower than monogastric species due to a limited capacity for hepatic fatty acid synthesis (Zammit and Corstorphine, 1982; Alaedin et al., 2021), altered *ACACA* expression has been reported with changes in hepatic lipid content in early-lactation cows (Du et al., 2018; Mann et al., 2018; Jia et al., 2019). Despite indications of increased fatty acid oxidation with Mon, we observed a tendency for increased *ACACA* expression in LS cows fed Mon relative to all other treatments (*P* = 0.14; 1.33 vs. 1.03, 1.27, and 1.12 for LS Con, HS Con, and HS Mon, respectively). The biological relevance of this finding is unclear given liver triglyceride content was not influenced by starch level and was decreased by Mon in multiparous cows in our companion paper (McCarthy et al., 2015b). Except for *ACACA* expression, changes in genes related to fatty acid metabolism were consistent with our previously reported results (McCarthy et al., 2015b).

The activity of *PC*, which is a key anaplerotic enzyme in the TCA cycle (Owen et al., 2002), is critical in providing a pool of OAA for gluconeogenesis (Aiello and Armentano, 1987; Figure 1). During early lactation it is likely that amino acids and lactate are supplying additional sources of OAA to maintain TCA cycle activity (Drackley et al., 2001). The mRNA expression of *PC* has been shown to increase during feed restriction in dairy cows (Velez and Donkin, 2005), and the increase in mRNA abundance for *PC* has also been strongly associated with an increase in *PC* activity (Greenfield et al., 2000). One of the key regulatory enzymes in the gluconeogenic pathway is *PCK1* (Croniger et al., 2002), and the *PCK1* gene promoter is positively regulated by propionate (Hazelton et al., 2008). In the current study, Mon supplementation in multiparous cows tended to increase *PC* (0.58 vs. 0.43; *P* = 0.13) and *PCK1* (1.07 vs. 0.76; *P* = 0.09) relative to multiparous controls, although there was no difference between primiparous animals (0.45 Mon vs. 0.48 Con, *PC*; 0.87 Mon vs. 0.95 Con, *PCK1*). These results support observations from our companion publication, which demonstrated an increased propensity to convert [1-^14^C]propionate to glucose in vitro in Mon-supplemented cows (McCarthy et al., 2015b). This increased removal of OAA from the TCA cycle to support gluconeogenesis would increase the need for additional OAA to condense with acetyl-CoA and generate ATP to maintain TCA cycle function, which is supported by increased *PC* expression.

Increased *PCK1* expression with Mon supplementation agrees with observations from Karcher et al. (2007), which found increased *PCK1* expression at d −14 and 1 relative to calving in cows fed monensin (300 mg/d) beginning −28 d before calving until calving. However, effects were no longer observed by 7 d postpartum (Karcher et al., 2007). In addition, others have reported no effect of early lactation propionate infusion on *PCK1* expression (Stocks and Allen, 2013), indicating variability in the response of *PCK1* expression to increased propiogenic substrate supply at the liver. Other reports suggest that *PCK1* expression is not upregulated until d 28 postpartum (Greenfield et al., 2000), indicating that perhaps other gluconeogenic enzymes are of greater importance during very early lactation. Interestingly, no independent effects of starch level were observed on *PC* and *PCK1* expression herein.

Although we hypothesized that increasing starch level and feeding monensin during the immediate postpartum period would increase hepatic gluconeogenesis by increasing the expression of gluconeogenic and fatty acid oxidation enzymes, absolute differences between treatments were relatively minimal. There were, however, interesting differences in the relative relationships between genes for cows fed diets of greater propiogenic potential. Correlation analysis performed for all gene expression variables are presented in Table 2. Overall, relationships were similar in directionality and magnitude between cows fed HS and LS and Con and Mon cows with a few exceptions. Cows fed Mon had a greater relationship between *ACACA* and *HMGCS2* than control (r = 0.56, *P* < 0.001, Mon; r = 0.15, *P* = 0.47, Con). Cows fed Mon also had no relationship between *HMGCS2* and *PC* (r = −0.08; *P* = 0.72), whereas Con cows had a positive relationship (r = 0.44; *P* = 0.03). Similarly for cows fed HS, there was no relationship between *HMGCS2* and *PC* (r = −0.06; *P* = 0.79), whereas LS cows tended to have a positive relationship (r = 0.35; *P* = 0.10). The absence of a relationship between PC and HMGCS2 in cows fed more propiogenic diets is consistent with propionate supply allowing for replenishment of TCA cycle intermediates during periods of increased glucose demand.

**Table 2:** Pearson's correlations for hepatic gluconeogenic and fatty acid gene expression between cows fed either high- or low-starch diets and control or monensin treatments in early lactation

Item^1^	*ACACA*		*CPT1A*		*HMGCS2*		*PC*		*PCK1*	
	Prop^2^	Con^2^	Prop	Con	Prop	Con	Prop	Con	Prop	Con
*ACACA*										
Mon,^3^ r	1.00	1.00	0.29	0.11	0.56	0.15	−0.10	−0.13	0.32	0.15
Mon, *P*			0.17	0.61	<0.01	0.47	0.63	0.53	0.12	0.47
Starch,^4^ r	1.00	1.00	0.03	0.27	0.37	0.26	−0.23	−0.01	0.08	0.34
Starch, *P*			0.89	0.21	0.07	0.22	0.27	0.97	0.69	0.11
*CPT1A*										
Mon, r			1.00	1.00	0.41	0.47	0.64	0.76	0.72	0.63
Mon, *P*					0.04	0.02	<0.01	<0.01	<0.01	<0.01
Starch, r			1.00	1.00	0.53	0.42	0.64	0.70	0.69	0.60
Starch, *P*					0.01	0.04	<0.01	<0.01	<0.01	<0.01
*HMGCS2*										
Mon, r					1.00	1.00	−0.08	0.44	0.34	0.78
Mon, *P*							0.72	0.03	0.10	<0.01
Starch, r					1.00	1.00	−0.06	0.35	0.35	0.72
Starch, *P*							0.79	0.10	0.08	<0.01
*PC*										
Mon, r							1.00	1.00	0.58	0.51
Mon, *P*									<0.01	<0.01
Starch, r							1.00	1.00	0.39	0.65
Starch, *P*									0.05	<0.01
*PCK1*										
Mon, r									1.00	1.00
Mon, *P*										
Starch, r									1.00	1.00
Starch, *P*										

1 *ACACA* = acetyl-CoA carboxylase α; *CPT1A* = carnitine palmitoyl-transferase 1A; *HMGCS2* = 3-hydroxy-3-methyl-glutaryl-CoA synthase; *PC* = pyruvate carboxylase; *PCK1* = cytosolic phosphoenolpyruvate carboxykinase.

2 Prop = propiogenic diet; Con = control diet.

3 Mon = where monensin treatment formulated to supplement 450 mg/d postpartum is the propiogenic diet, and control treatment formulated to supplement 0 mg/d monensin is the control.

4 Starch = where the high-starch diet (26.2% starch) is the propiogenic diet, and the low-starch diet (21.5% starch) is the control treatment.

Although results on expression of gluconeogeneic and fatty acid oxidation enzymes at 7 d postpartum were minor, overall, results support changes in performance and energetic metabolism reported in our companion papers, indicating that cows fed diets of different starch content in early lactation with Mon supplementation throughout the transition period had alterations in hepatic gene expression consistent with increased hepatic propionate supply.
